# Stable and Reversible Photoluminescence from GaN Nanowires in Solution Tuning by Ionic Concentration

**DOI:** 10.1186/s11671-021-03473-7

**Published:** 2021-03-11

**Authors:** Anh Thi Nguyen, Ya-Wen Ho, Wei-Cheng Yu, Hsiao-Wen Zan, Hsin-Fei Meng, Yi-Chia Chou

**Affiliations:** 1 Institute of Physics, National Yang Ming Chiao Tung University, Hsinchu, 30010 Taiwan; 2 Department of Photonics, National Yang Ming Chiao Tung University, Hsinchu, 30010 Taiwan; 3 Department of Electrophysics, National Yang Ming Chiao Tung University, Hsinchu, 30010 Taiwan

**Keywords:** GaN nanowires, Photoluminescence response, Ionic concentration, Physical interaction, Reversibility

## Abstract

We report response of photoluminescence (PL) from GaN nanowires without protection in solutions. The distinct response is not only toward pH but toward ionic concentration under same pH. The nanowires appear to be highly stable under aqueous solution with high ionic concentration and low pH value down to 1. We show that the PL has a reversible interaction with various types of acidic and salt solutions. The quantum states of nanowires are exposed to the external environment and have a direct physical interaction which depends on the anions of the acids. As the ionic concentration increases, the PL intensity goes up or down depending on the chemical species. The response results from a competition of change in surface band bending and charge transfer to redox level in solution. That of GaN films is reported for comparison as the effect of surface band bending can be neglected so that there are only slight variations in PL intensity for GaN films. Additionally, such physical interaction does not impact on the PL peaks in acids and salts, whereas there is a red shift on PL when the nanowires are in basic solution, say NH4OH, due to chemical etching occurred on the nanowires.

**Supplementary Information:**

The online version contains supplementary material available at 10.1186/s11671-021-03473-7.

## Introduction

Crystalline GaN has been chosen as a promising semiconductor material with a wide direct bandgap of 3.39 eV. It has many good material properties which have been applied to extensive applications, such as blue/ultraviolet light emission diodes [1, 2], optoelectronics [3, 4], high-temperature/high-power devices [5, 6], and field effect transistor [7, 8]. Compared to bulk material, one-dimensional nanostructures exist intrinsically efficient lattice relaxation [9]. Thus, they can be grown with less crystal defects [10], and this composes the main benefit where photoluminescence (PL) emission and electrical properties are affected by these defects. Long nanowires provide larger surfaces to be utilized, which could be a great usage in sensor and chemistry applications [11, 12]. Besides, GaN is stable and does not strict to vacuum, which enables the applications in chemical environments.

Photoluminescence is one of the most commonly used properties to study the quantum states of a material [13, 14], as PL comes from the radiative decay of the excited states. For solid-state materials, the interaction of PL with the surrounding chemical conditions provides a way to probe the excited states. Such surface interaction can also be used for chemical sensors and imaging [15, 16]. However, for most materials, the PL is degraded under a reactive environment because it is easily quenched by the surface defect states or by the transfer of photo-generated carriers to the ionic species in the solution [17, 18]. In particular, PL is unstable for most organic and inorganic semiconductors in the aqueous solution [19–21]. Consequently, for the fluorescent inorganic semiconductor quantum dots, a core–shell structure is often required to protect the light-emitting quantum states [21–25]. Thus, the interaction between the chemicals in solution and the quantum state becomes weak due to the protection.

Optical pH sensors were proposed which overcame problems such as signal drift, parasitic photocurrent effects, and the encapsulated electric contact stability in chemically aggressive liquids, though they could not cover a wide pH range [26]. The luminescence properties of coated and uncoated inorganic nanoparticles could respond to pH variations in a wider range [27, 28], but electric potential of free nanoparticles cannot be controlled prohibiting a quantitative analysis of the pH response. The excellent optical properties of group III nitride nanowires [29, 30] enable the use of pH and bias response to the PL intensity as detection signal [31]. The position of the band edges in group III nitride materials with respect to the redox levels of water attracts attention for the application in photocatalytic water splitting by charge transfer across the group III nitride-material/electrolyte interface [32–34]. The efficiency of this process has been shown to be enhanced if nanowires instead of planar electrodes are used [35].

So far most of the PL study on GaN nanowires is done in air. There are few reports discussing PL in solution. The ions may change the photo-carrier recombination process. Optical excitation of nanowires in solution is related to potential applications like water splitting and ion sensor.

In this work, we study the response of PL from GaN nanowires, which contain highly sensitive GaN surface [35–38], immersed in water including acid, base, and salts without any protection. The setup, distinct from typical setup for electrochemical reaction [39–43], is less complicated without plating electrochemical terminals. The PL response is measured and correlated with ion concentration. The quantum states of nanowires are exposed to the external environment and have a direct physical interaction. We found that PL mostly rises with ionic concentration for nanowires, but decreases for films. We show that the PL dependence on ions has a reversible interaction with various types of acid and salt solutions. Previously, there was a report which used the standard phosphate buffered saline with a phosphate concentration of 0.01 M to keep it at constant high ionic concentration so only the pH values were changed in the measurements and the PL of nanowires depended on the pH value only [31]. In our work, there is no background buffer solution and the ionic concentration is variable. The PL responds toward ions instead of merely toward pH values. It turns out that the PL is not just controlled by the pH value but also depends on the concentration of anions of the acids. The reaction mechanism reported here is different from electrochemistry which requires electrodes for measurements, and the ion concentration in our experimental setup changes with pH which chiefly contributes PL. The trends can be interpreted by the competition of two effects of the ions: the carrier transfer to redox level and the change of the depletion region.

From an application point of view, a stable PL response under harsh chemical environment can be applied to monitor the waste or polluted water over a long time. If a piece of GaN nanowires is immersed in such water, its PL response can be obtained from external optical excitation and fluorescence collection. The advantage of GaN nanowires is that it has only physical interaction but no chemical interaction due to its superior stability in extreme conditions. The conventional test paper or electrochemical sensor cannot work in such conditions over a long period. Besides, the three electrochemical terminals are not necessary in our experimental setup to maintain the electrolyte potential. This makes the setup much simpler. There is no chemical reaction; a clear picture of the photo-carrier relaxation process is established for the important material of GaN nanowires in electrolyte solution. Such picture may benefit the future development of new GaN applications like ion sensor or water splitting.

## Methods

### Synthesis of GaN Nanowires and Films

The growth of GaN nanowires via VSS mechanism and GaN films were fabricated in a hydride vapor phase epitaxy (HVPE) system [44, 45], where the vacuum level is at 1 atmosphere. The precursor gases were ammonia (NH_3_) and gallium chloride (GaCl) formed by flowing HCl gas diluted with nitrogen through molten Ga at 850 °C. For nanowire growth, Ni was selected as the catalyst and the growth took place at 880 °C with V/III = 20 and carrier gas N_2_ of 400 sccm. GaN nanowires then grew when the two precursor gases, GaCl (as the source of Ga) and NH_3_ (as the source of N), met and reacted near the samples at 650–950 °C. The GaN nanowires were m-axis oriented [45]. Note that the Ni-Ga catalysts were etched away by HCl from the flow and the side product of reactions during growth [46]. The GaN thick film with c-orientation was grown on sapphire in the HVPE system at a growth temperature of 1050 °C. The thickness of GaN films is 300 ± 10 μm.

### Sample Preparation

The GaN samples were stored in low vacuum (~ 10–1 Torr) after growth and were treated using HF vapor to remove possible oxide before PL measurements.

### PL Measurement

The setup of the PL property measurements is shown in Additional file 1: Figure S1. Helium–cadmium (He–Ca) laser is used as the excitation light source for continuous-wave output at a wavelength of 325 nm. The function of fiber was to collect photon emission of PL and connect to iHR 550 Imaging Spectrometer designed for spectral measurements. iHR 550 was an automated, triple-grating spectrometer.

The entrance and exit slits in the system played a key role. Particularly, the wider they were, the lower the resolution of the PL spectrum would be. However, if the slits were too narrow, noises could significantly affect the signals. In this measurement, we used a 1200 grooves/mm grating and the slit was 0.2 mm with the aim of obtaining excellent resolution. Photomultiplier tube was the detector with the power supply (950 V). In order to allow the laser to focus on the sample, the measurements had to use three dichroic mirrors and two focus lens. After concentrating, the spot diameter was about 0.3 mm and the power density attained 21 W/cm^2^ on the surface of the sample. The photograph of the experimental setup is shown in Additional file 1: Figure S2.

### Morphology Observation

The morphology of the GaN nanowires was examined using a scanning electron microscope (SEM; JEOL-6700F SEM).

## Results and Discussion

### PL Response from GaN Toward Ion Concentration in Acidic and Salt Solutions

**Fig. 1:**
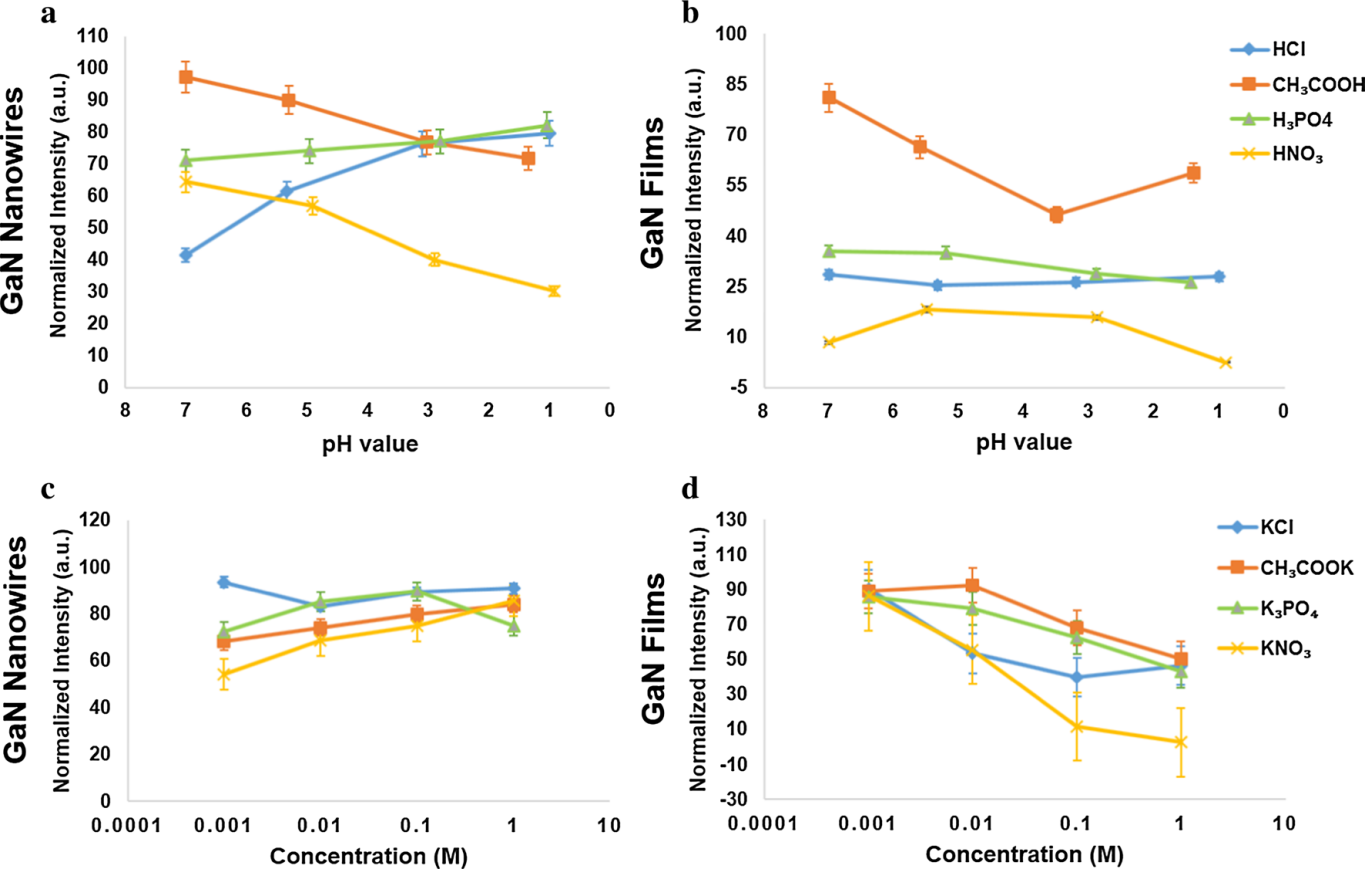
The PL intensity distribution in different pH values of acidic solutions and salt solutions at different concentrations. He–Ca laser is used here and below as the excitation light source for continuous-wave output at a wavelength of 325 nm. **a** The PL response from GaN nanowires with diameters ranging between 60 and 80 nm in acidic solutions. **b** The PL response from GaN films with ~ 300 μm in thickness in acidic solutions. **c** The PL response from GaN nanowires in salt solutions with different concentrations. **d** The PL response from GaN films in salt solutions with different concentrations

Figure 1a, b illustrates the influence of the pH values ranging from 1 to 7, using acidic solutions and DI water, on GaN nanowires and GaN films. They show that the PL intensity with different pH values has two remarkable trends for different acidic solutions. In particular, the PL intensity of GaN nanowires in hydrochloric acids (HCl) increases significantly with pH varying from 7 to 1 and that in phosphoric acid (H_3_PO_4_) increases slightly, whereas the intensity decreases sharply when the GaN nanowires are nitric acid (HNO_3_) and acetic acid (CH_3_COOH) with pH values decreasing from 7 to 1 as shown in Fig. 1a. Similarly, in Fig. 1b, it presents the results of the PL intensity of GaN films changes in these acidic solutions. The PL response experiences a decrease in CH_3_COOH from neutral to lower pH and gentle downward or stable trends in the rest of acidic solutions. The most striking difference in comparison with the effect on GaN nanowires and thick films is that the PL intensity of GaN nanowires remains consistently increasing or decreasing, but that of GaN films is relatively stable as either they go down or the variation stays within a range. Note that the ion concentrations of acidic solutions are different corresponding to pH values. The tests of the samples in salt solutions for which the pH are 7 but with different ion concentrations are performed to verify the response toward ions at the constant pH.

To confirm the tendency with ion concentration, we investigated the effects of salt solutions with different concentrations on PL intensity in terms of two kinds of materials: GaN nanowires and GaN films as shown by the line graphs in Fig. 1c, d. The PL intensity of GaN nanowires witnesses a steady increase in potassium acetate (CH_3_COOK) and potassium nitrate (KNO_3_) along with the rise in salt concentration from 0.001 to 1 M. In contrast, that in potassium chloride (KCl) goes down from 0.001 to 0.01 M but gradually moves up from 0.01 to 1 M. That in tripotassium phosphate (K_3_PO_4_) has a significant rise from 0.001 and 0.1 M, while the slope declines from 0.01 to 0.1 M. The intensity goes down when the concentration goes up to 1 M. Considering Fig. 1d for GaN films, all the slopes show downward trends in the four types of salts with increase in salt concentration, which is different from that of GaN nanowires. The results are consistent with that in acidic solutions where GaN films have downward trends on PL intensity, but the intensity can be either move up or down with pH change. As the ionic concentration increases, the PL from nanowires goes up or down with slopes depending on the chemical species. Thus, ionic species and concentrations play critical roles on PL.

**Fig. 2:**
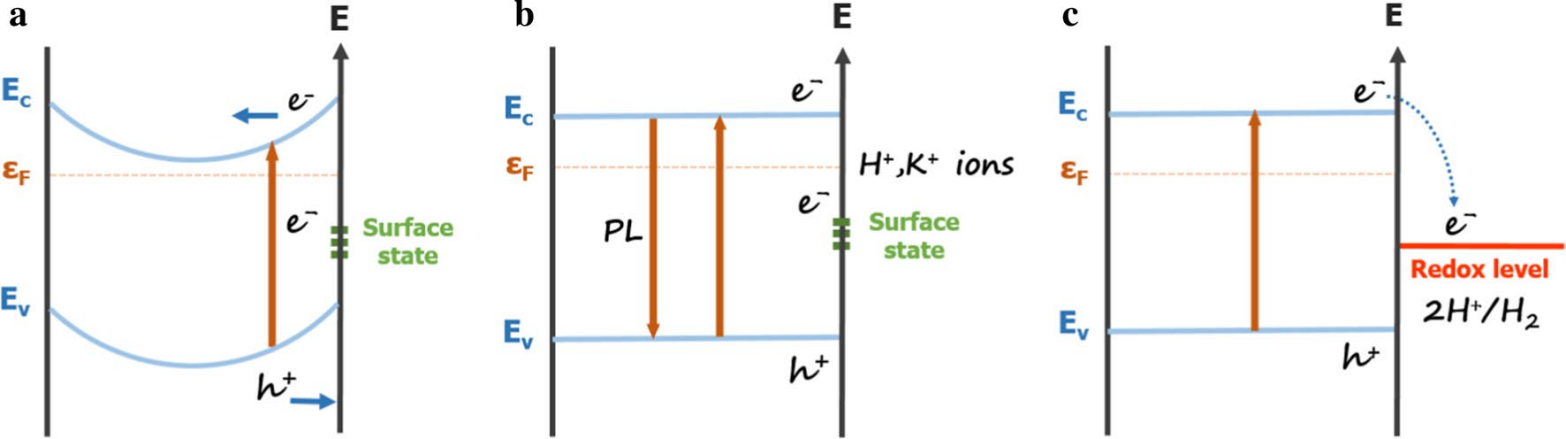
Schematic illustration of the effect of ion concentration on photoluminescence intensity. *E* is energy. *E*_c_, *E*_v_, and *ε*_F_ are conduction band edge, valance band edge, and Fermi level respectively. **a** Surface depletion region caused by the trapped electrons in the surface states. **b** Depletion region neutralized by the attachment of positives ions H^+^ or K^+^. **c** Electron transfer to the redox level of H^+^

The trends probably result from the combination of two factors that affect the PL. The first one is the reduction of the depletion region by the ionic attachment on the surface. The second one is the electron transfer to the redox levels of the ions. To understand the two non-identical trends of PL intensity in different acidic solutions and salt solutions for GaN nanowires and GaN films, Fig. 2 shows schematically two mechanisms by which the ion concentration controls the PL intensity: (A) depletion region reduction and (B) charge transfer to redox level [31, 47].

From the modeling of interface of semiconductor and electrolyte solution [47], we understand that ions are distributed only between the solution and the surface of the solid, that is, there are charged layers at both GaN and solutions. The potential at the interface was then adjusted which caused band bending. Photocatalytic water splitting from powdered GaN under light irradiation results from the band energy scheme of GaN where the conduction band edge of GaN is positioned 0.5 V higher than the redox potential of H + /H_2_ [30]. GaN nanowires exhibit better photocatalytic activity because of the large surface area which enhanced photocatalytic activity in the acid pH region [38]. Because of the surface states at the bandgap, the electrons will fill in the gap state and the Fermi level is pinned over there [34, 43, 48]. The band bending is shown in Fig. 2a. Near the surface, there is a depletion region. Because of the strong electric field in the depletion region, the photo-generated electrons and holes will be driven to opposite directions and the recombination is prevented. When there is a high concentration of ions in the solution, the ions may attach to the surface to neutralize surface charge density and the depletion is reduced. Such attachment and detachment of ions alters the charge distribution in the bandgap in pH sensors [49]. Once the energy band becomes nearly flat, the electron–hole recombination is restored and PL is enhanced as also shown in Fig. 2a.

Because of the large surface area of nanowires, the depletion region mechanism is more important in the nanowires than in the films. In fact, the small nanowires are fully depleted [34]. In mechanism (B), the ions play a different role to affect the PL as shown in Fig. 2b. For GaN nanowires, the redox levels of H^+^ and OH^−^ are within the bandgap [36, 50–52]. The photo-generated carrier can, therefore, transfer to the redox level instead of recombining. For example, the reaction between protons and the photo-carriers 2H^+^ + 2e^−^ → H_2_ may take place. Because the depletion region only takes a small fraction of the films where the majority of the film is not depleted, mechanism (A) is relatively unimportant. Hence, the charge transfer mechanism dominates for the films. Note that as ionic concentration increases, for mechanism (A) the PL is enhanced, whereas for mechanism (B) PL is reduced. For GaN nanowires, the PL is determined by the competition of (A) and (B). For GaN films, the PL is determined by (B) mostly because of the small fraction of depletion region from its limited surface. This explains why GaN nanowires have either increasing or decreasing PL trends with increasing ion concentration, but GaN films have only decreasing or nearly constant PL.

In addition to the solution condition, the plane orientation and polarity of the two forms of GaN are dissimilar. The GaN films are c-plane with Ga-polarity, but growth front of the nanowires is non-polar m-plane. The surface area (the sidewall) is larger in nanowires which exhibit a variety of sets of crystallographic planes if assuming the cross section is nearly circular. The polarity may partially contribute to the pH-dependent PL from GaN nanowires and films.

### Effect of Surface Area on PL Response

**Fig. 3:**
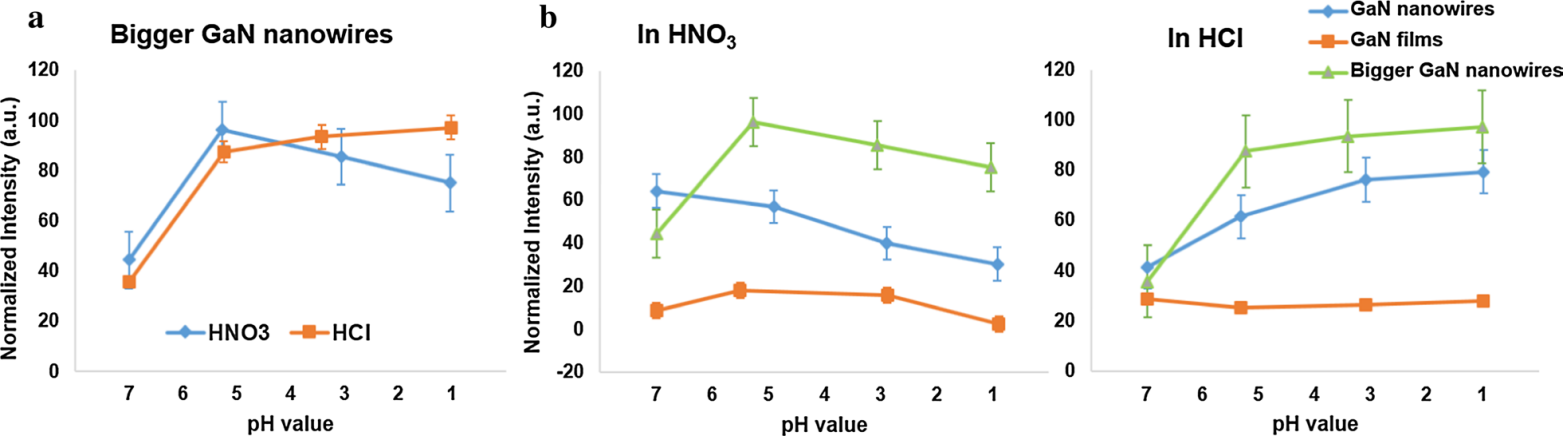
Comparison of PL intensity from different sizes and dimensions of GaN. **a** The PL intensity distribution in various pH values of acidic solutions on bigger GaN nanowires with diameters of ~ 200 nm. **b** A comparison of PL intensity for GaN nanowires, GaN films and bigger GaN nanowires in HNO_3_ and HCl

Regarding the morphology of GaN, e.g., nanowire and film, we also investigated the PL of bigger nanowires for comparison. The diameters of such nanowires are ~ 200 nm, while the typical nanowires are ~ 60–80 nm. Figure 3a shows how HNO_3_ and HCl affect the PL intensity of bigger GaN nanowires under variation of pH from 7 to 1. When they are immersed in HNO_3_, PL intensity reaches a peak at pH = 5.27. On the contrary, it has a remarkable jump of PL intensity in pH = 5.27 of HCl and steadily increases when pH goes down. In Fig. 3b, we make a comparison of PL intensity of three kinds of samples (nanowires, films, and bigger nanowires) in nitric and hydrochloric acidic solutions. The PL intensity distribution of nanowires with typical sizes and bigger nanowires has downward patterns with lowering pH in HNO_3_ but upward in HCl. In other words, the two sizes of nanowires perform similarly on PL response in acidic solutions and it follows the mechanisms discussed in Fig. 2. Apparently, the PL intensity of GaN films in both HCl and HNO_3_ is relatively stable and that in HCl is approximately constant.

**Fig. 4:**
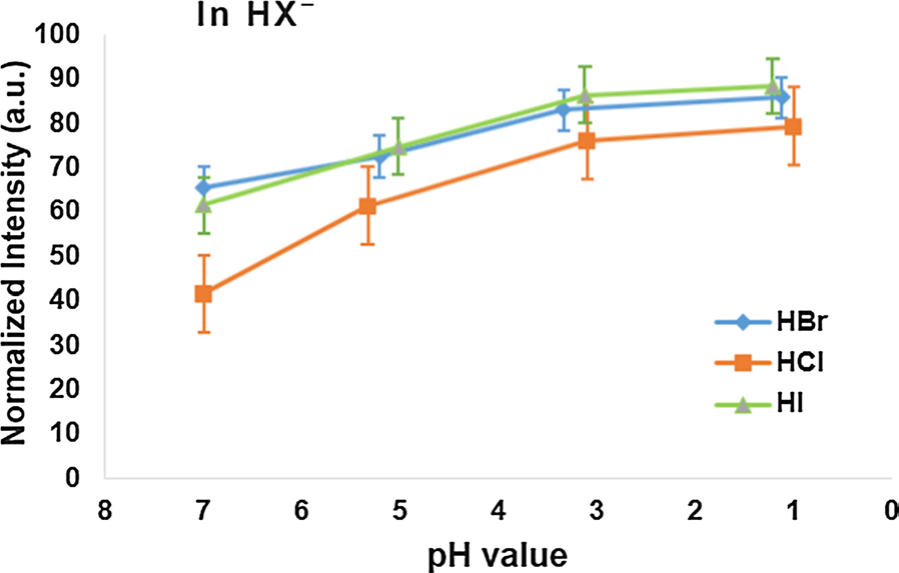
The PL intensity distribution of GaN nanowires under pH variations in three hydrohalic acidic solutions

Furthermore, the PL intensity of GaN nanowires in hydrohalic acids, e.g., hydrobromic acid (HBr), hydrochloric acid (HCl), and hydriodic acid (HI), fluctuates gradually with an upward movement when decreasing pH values from 7 to 1 as shown in Fig. 4.

### Reversibility Interaction

**Fig. 5:**
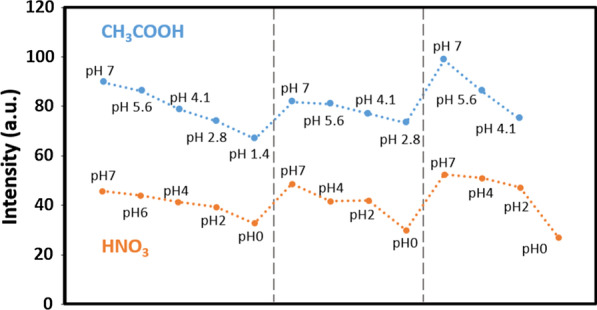
Reversibility of PL intensity of GaN nanowires in CH_3_COOH and HNO_3_ with various pH values

To evaluate the repeated use for applications, the tests were repeated with nanowires in solutions from neutral pH to low pH. The samples were refreshed by DI water between each test as noted by pH 7 in Fig. 5. The PL intensity moved down when pH went lower, but it restored after DI water cleaning on samples. Such reversibility of the PL intensity under changing pH from GaN nanowires in acidic solutions enables long-term applications in acidic chemical environments. Besides, the PL peaks at which nanowires were immersing in acidic solutions and upon removal of the solutions keep at ~ 3.4 eV (Additional file 1: Fig. S1–S4). It confirmed that the microstructures of the GaN nanowires were not damaged during the solution treatments.

### Effect on Crystal Quality

**Fig. 6:**
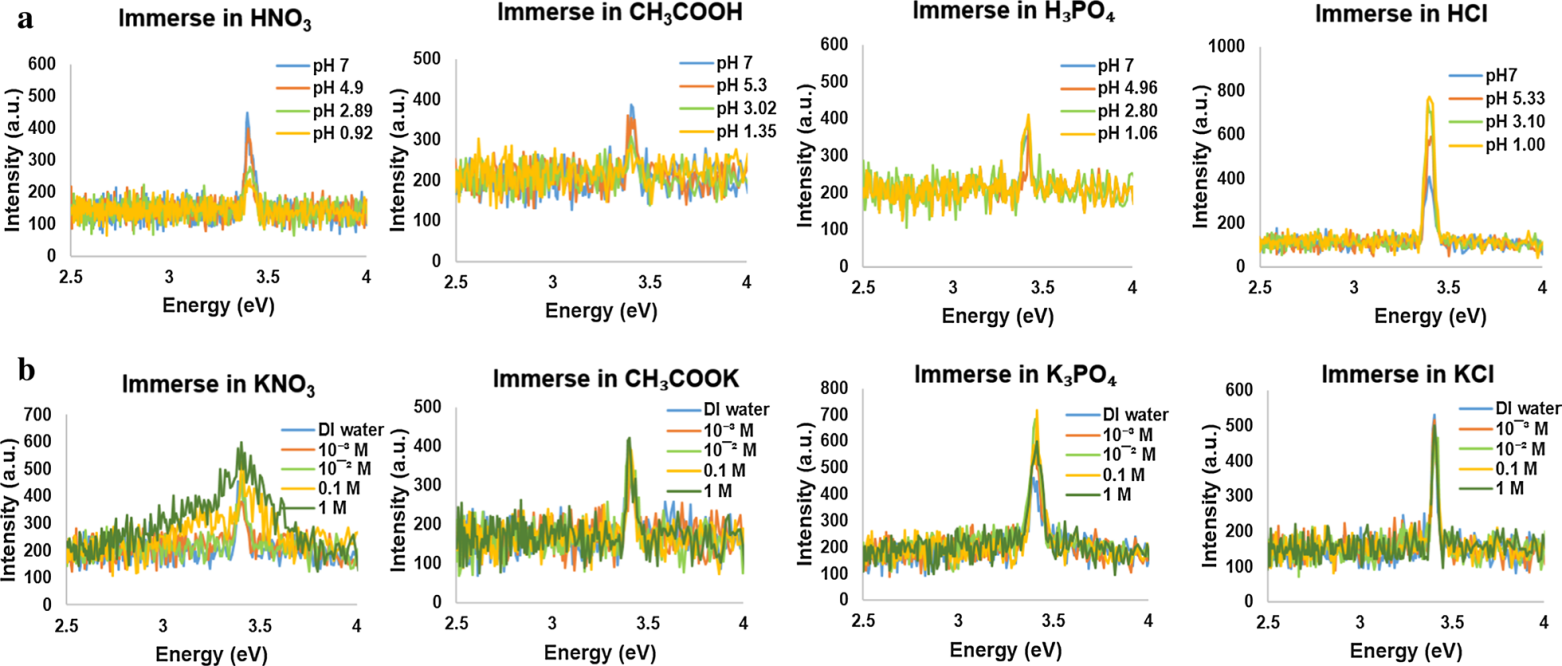
PL spectra when GaN nanowires immerse in solutions of different pH or concentrations. **a** PL spectra of GaN nanowires in acidic solutions and **b** PL spectra of GaN nanowires in salt solutions

Figure 6 provides the details of PL spectra about immersion of GaN nanowires in the four acidic solutions (Fig. 6a) and salt solutions (Fig. 6b). It depicts the variation of PL intensity with pH values or ionic concentrations, but the main peaks are still at ~ 3.4 eV. The wide direct bandgap of 3.4 eV of GaN [46, 53] maintains in those acidic solutions which suggests that it exhibits physical interaction instead of chemical reactions. The trends of intensity with pH values are discussed in Fig. 1. The stable main peak at ~ 3.4 eV (Fig. 6 and Additional file 1: Fig. S3–S7) indicates the microstructure of GaN nanowires maintains after acidic solution immersion.

Note that the surface oxide could contribute to yellow luminescence (YL) which is emitted from surface states associated with the gallium oxide [54] that decorates the free surface and possibly also the substrate interface and internal grain boundaries. From our PL spectra, the YL is not significant which indicates that Ga oxide was mostly removed by the HF vapor treatment before measurement. In addition, HCl can etch the surface oxide layer as well to eliminate the oxide contribution to PL.

**Fig. 7:**
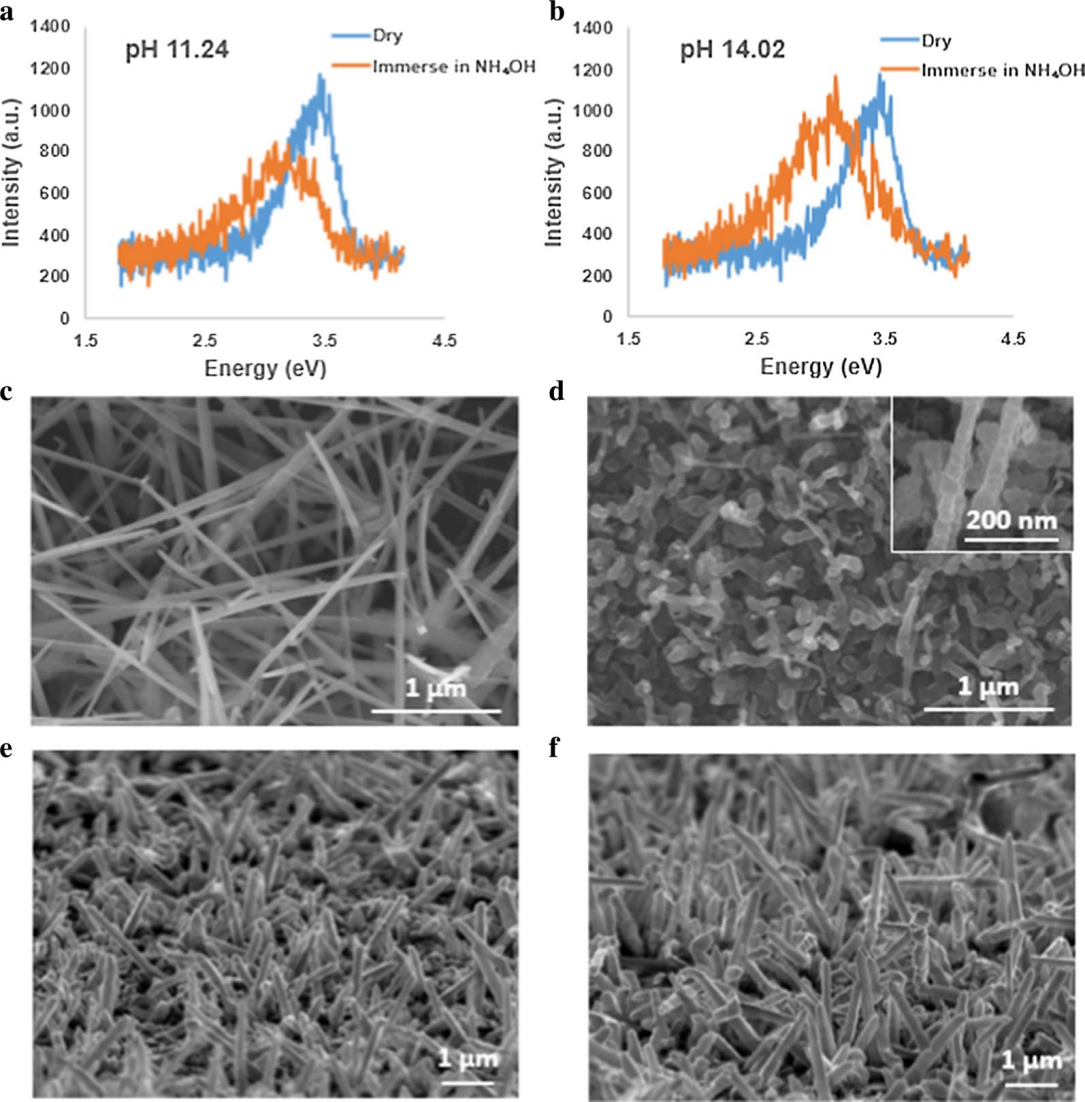
The PL spectra of GaN nanowires in NH_4_OH and the corresponding SEM images. **a** Signals when nanowires are in NH_4_OH at pH = 11.24. **b** Signals when nanowires are in NH_4_OH at pH = 14.02. **c** SEM image of the as-grown GaN nanowires. **d** The GaN nanowires after immersing in NH_4_OH solution for 20 min. **e** SEM image of the as-grown GaN nanowires. **f** The nanowires after the tests of immersing them into the acidic solution, HCl, for 20 min

Despite acids, we also investigated the impact of basic solution, e.g., ammonium hydroxide solution (NH_4_OH), on GaN nanowires for comparison through the PL response. The PL peaks of as-grown GaN nanowires were located at ~ 3.4 eV, while the peaks shifted when immersing them in NH_4_OH solution. The PL spectra for pH = 11.24 (Fig. 7a) and 14.02 (Fig. 7b) showed red shifts on peaks to ~ 3.10 eV. The shift suggested the degradation in GaN microstructures. The irreversible reaction (Additional file 1: Fig. S6) might be caused by chemical reactions on the GaN structures where the GaN nanowires were damaged (Fig. 7c, d). Structural defects were generated on the surface of the GaN nanowires which could alter the surface density of states, thus the PL emission. Upon removal of such solutions, the peaks stayed at 3.10 eV (Additional file 1: Fig. S6) which implies that the change was permanent and the tests were irreversible in NH_4_OH solution. In comparison, the morphology of the nanowires remained without visible surface etching in acidic solutions, say HCl, as shown in Fig. 7e, f.

Oxygen molecules are known to affect the PL of organic molecules. The PL comes mostly from spin singlet exciton. The singlet exciton could be reduced to non-emissive triplet exciton by the excitation of the oxygen molecules from the spin triplet state to the singlet excited state [55, 56]. In GaN, there is little energy difference between the singlet and triplet electron–hole pair. The presence of oxygen molecules is not expected to form a non-radiative decay channel and affect the PL. In all of our experimental conditions, there is a saturated oxygen level in the solution. The trend of PL variation for various ion concentration is therefore not due to the oxygen level.

## Conclusion

We have demonstrated the stability and the response to PL from GaN nanowires immersing in different kinds of acidic solutions at varying pH corresponding to different ion concentration, and in salt solutions with fixed pH but different concentrations. The PL response was measured and correlated with ion concentration. In both types of solutions, the PL responses from GaN nanowires were either increasing or decreasing. In comparison, the PL responses were relatively stable or slightly decreasing from GaN films. The PL responses were distinct from nanowire and film of GaN that resulted from the different band structures of them. The competition of change in surface band bending and charge transfer to redox level in solution were considered, where nanowire could be fully depleted from its large surface areas, but the depletion fraction was small and negligible for films where there were only slight variations in PL intensity. It turned out that the PL responds toward ions instead of merely toward pH values as the response of PL intensity in salt solutions and HX^−^ solutions confirmed the results. Such physical interaction did not impact on the PL peaks in acids and salts, whereas there was a red shift on PL when the nanowires were in basic solution, say NH_4_OH, due to chemical etching occurred on the nanowires. We showed that the PL had a reversible interaction with those acid and salt solutions. The stable and reversible PL response of GaN nanowires in those acidic solutions enables the potential application in such harsh chemical environments.

## Supplementary Information

**Additional file 1: Fig. S1–S2.** Experimental setup and procedure of PL measurement. **Fig. S3–S4.** PL spectra from GaN nanowires immersing in salts or acids and after removal of the solutions. **Fig. S5–S6.** PL spectra from GaN films immersing in acids or salts and after removal of the solutions. **Fig. S7.** PL spectra from GaN nanowires immersing in HBr, HCl, and HI. **Fig. S8.** The shift of PL of GaN nanowires in NH4OH.

## Data Availability

All data generated or analyzed during this study are included in this published article.

## Abbreviations

PL: Photoluminescence
HVPE: Hydride vapor phase epitaxy
NH_3_: Ammonia
GaCl: Gallium chloride
He–Ca: Helium–cadmium
HCl: Hydrochloric acids
H_3_PO_4_: Phosphoric acid
HNO_3_: Nitric acid
CH_3_COOH: Acetic acid
CH_3_COOK: Potassium acetate
KNO_3_: Potassium nitrate
KCl: Potassium chloride
K_3_PO_4_: Tripotassium phosphate
HBr: Hydrobromic acid
HI: Hydriodic acid
YL: Yellow luminescence
NH_4_OH: Ammonium hydroxide solution
