# Supplementary material for: Stable and Reversible Photoluminescence from GaN Nanowires in Solution Tuning by Ionic Concentration
Source: Nanoscale Res Lett. 2021 Mar 11;16:45. doi: 10.1186/s11671-021-03473-7 (PMC7952484; doi:10.1186/s11671-021-03473-7)
Supplement: Supplementary file 1 — Additional file 1: Fig. S1–S2. Experimental setup and procedure of PL measurement. Fig. S3–S4. PL spectra from GaN nanowires immersing in salts or acids and after removal of the solutions. Fig. S5–S6. PL spectra from GaN films immersing in acids or salts and after removal of the solutions. Fig. S7. PL spectra from GaN nanowires immersing in HBr, HCl, and HI. Fig. S8. The shift of PL of GaN nanowires in NH4OH. [file 11671_2021_3473_MOESM1_ESM.docx]

Supporting Information

Stable and Reversible Photoluminescence from GaN Nanowires in Solution Tuning by Ionic Concentration

*Anh Thi Nguyen, Ya-Wen Ho, Wei-Cheng Yu, Hsiao-Wen Zan, Hsin-Fei Meng,^*^ and Yi-Chia Chou^*^*

**
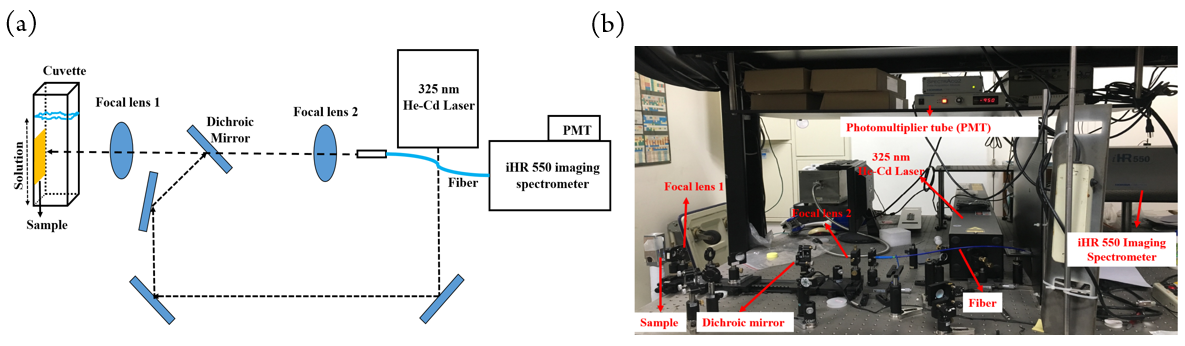
**

**Figure S1.** Experimental setup. (a) Schematic representation for PL measurement system. (b) Photo of PL measurement system.

**
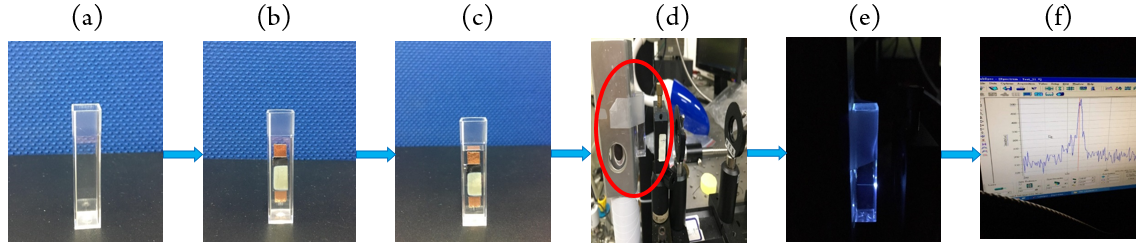
**

**Figure S2.** Procedure of PL measurements. (a) Dried cuvette. (b) Sample inside the cuvette. (c) Immerse in solutions. (d) Fix the cuvette on the setup. (e) Turn on the laser beam. (f) Obtain the PL spectrum.

**
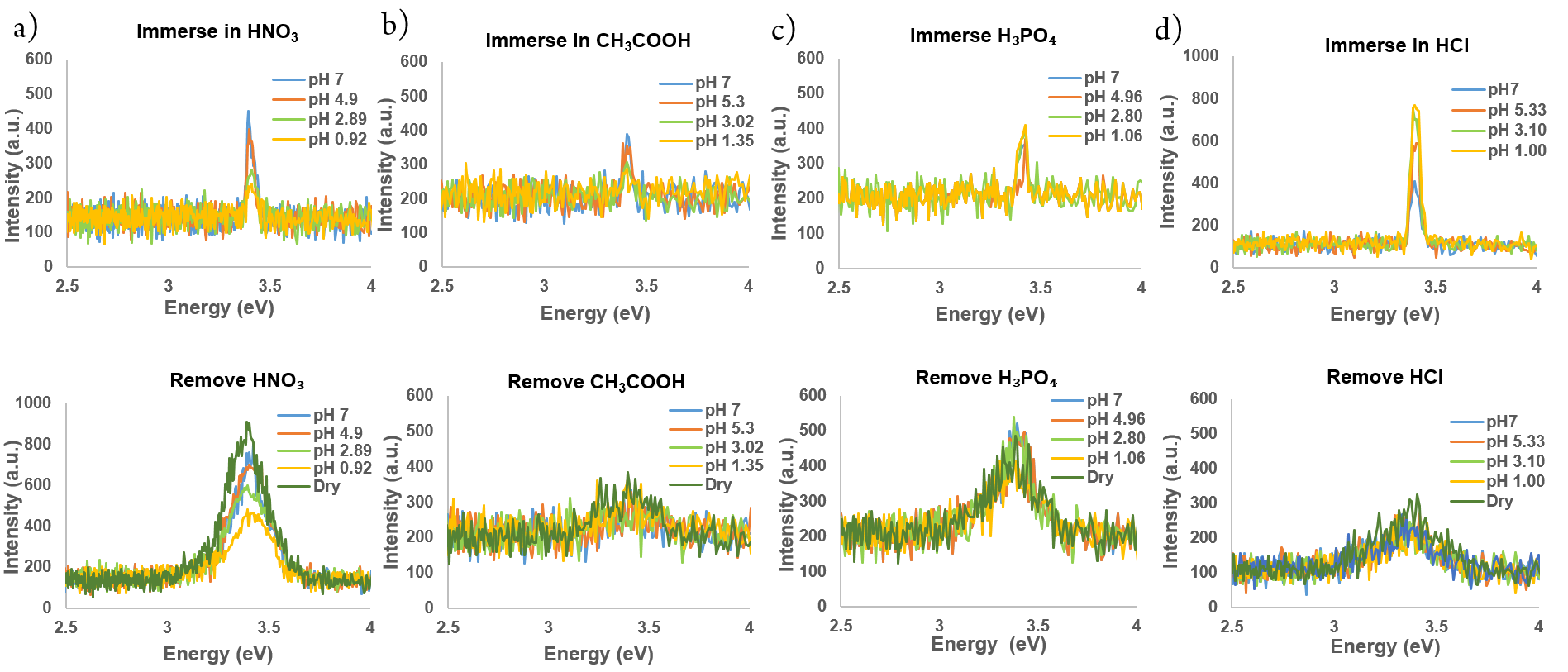
**

**Figure S3**. PL spectra when nanowires are immersing in acids and after removal of acids: (a) nitric acid (HNO₃), (b) acetic acid (CH₃COOH), (c) phosphoric acid (H₃PO₄), and (d) hydrochloric acid (HCl).


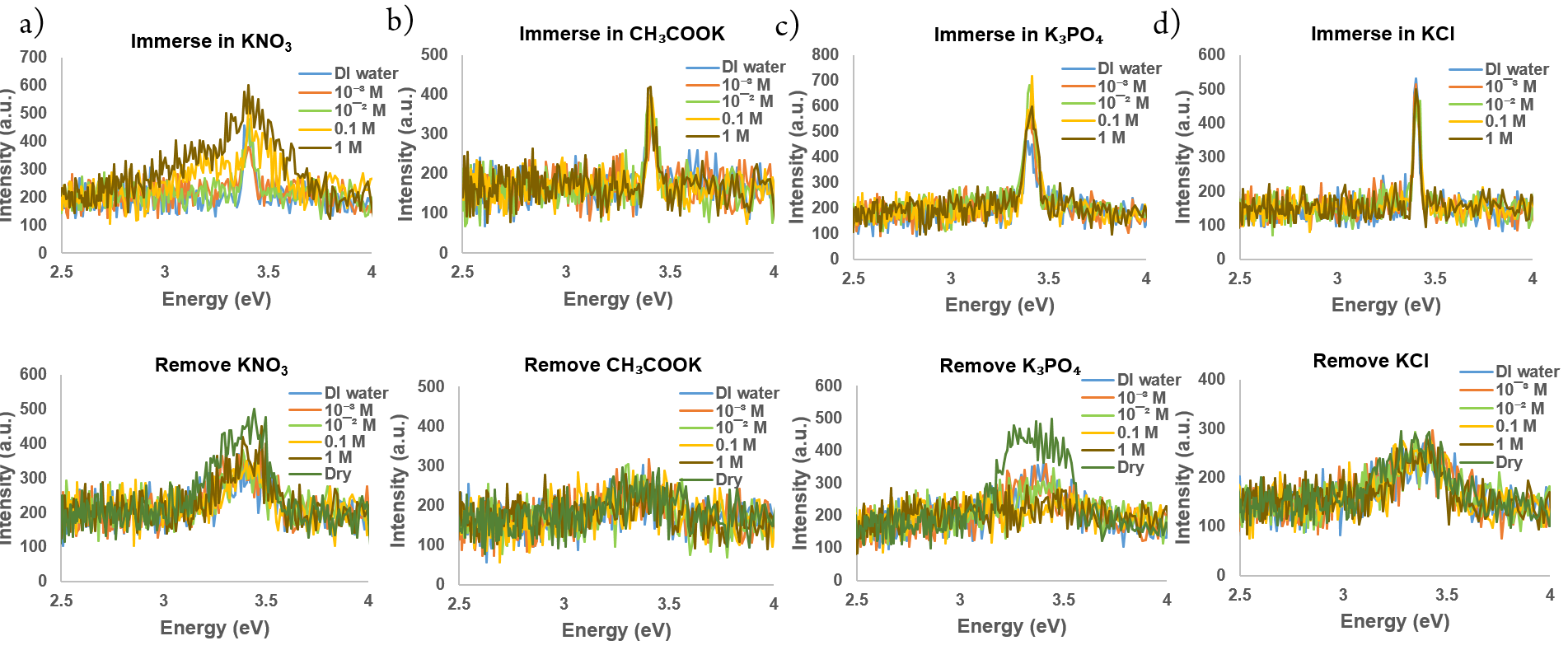


**Figure S4.** PL spectra when GaN nanowires are immersing in salts and after removal of salts: (a) potassium nitrate (KNO₃), (b) potassium acetate (CH₃COOK), (c) tripotassium phosphate (K₃PO₄), and (d) potassium chloride (KCl).


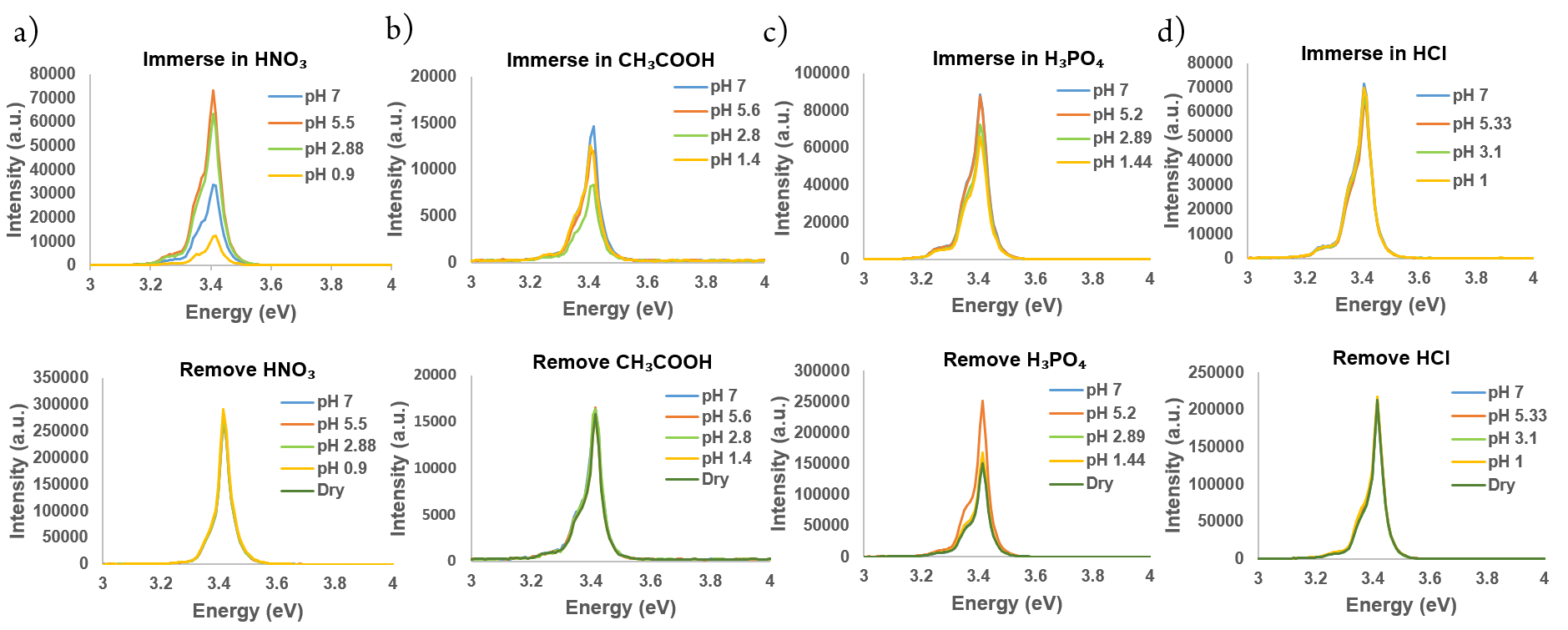


**Figure S5.** PL spectra when GaN films are immersing in acids and after removal of acids: (a) nitric acid (HNO₃), (b) acetic acid (CH₃COOH), (c) phosphoric acid (H₃PO₄), and (d) hydrochloric acid (HCl).


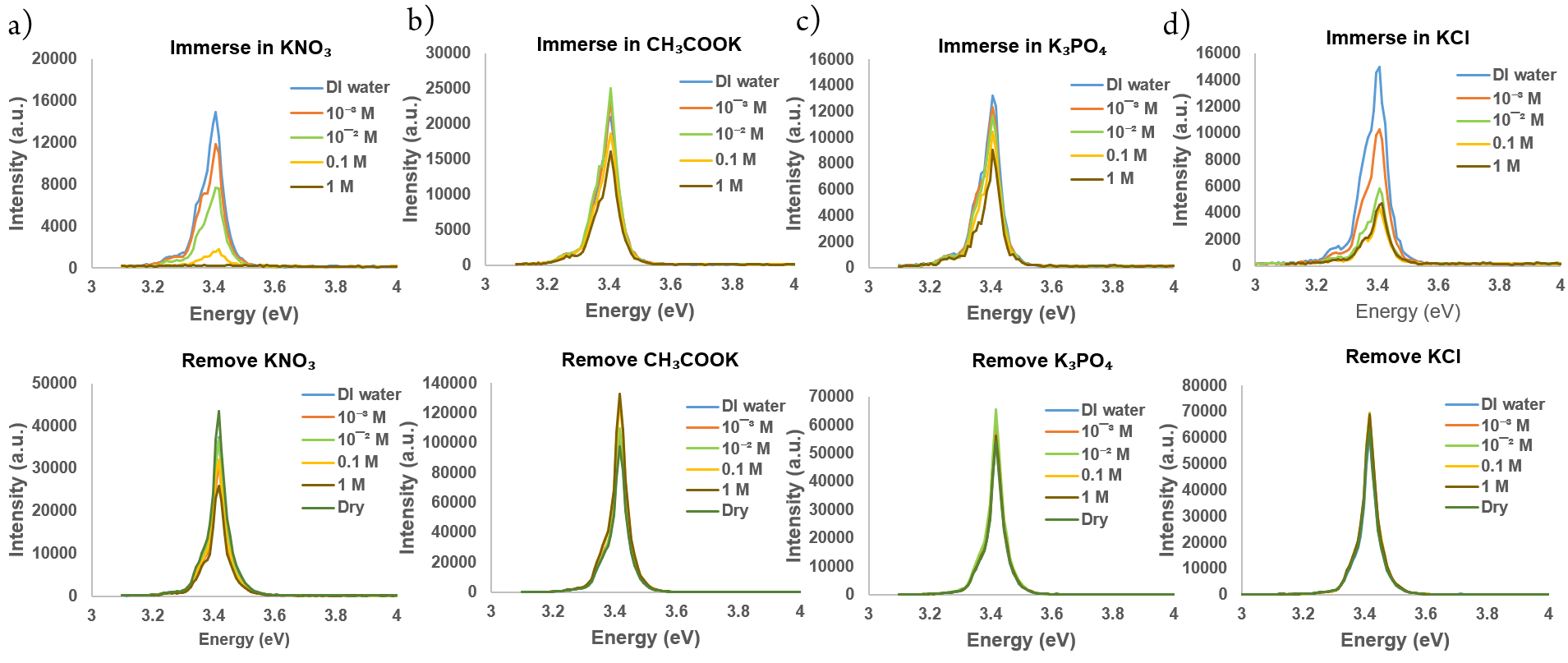


**Figure S6**. PL spectra when GaN films are immersing in salts and after removal of salts: (a) potassium nitrate (KNO₃), (b) potassium acetate (CH₃COOK), (c) tripotassium phosphate (K₃PO₄), and (d) potassium chloride (KCl).


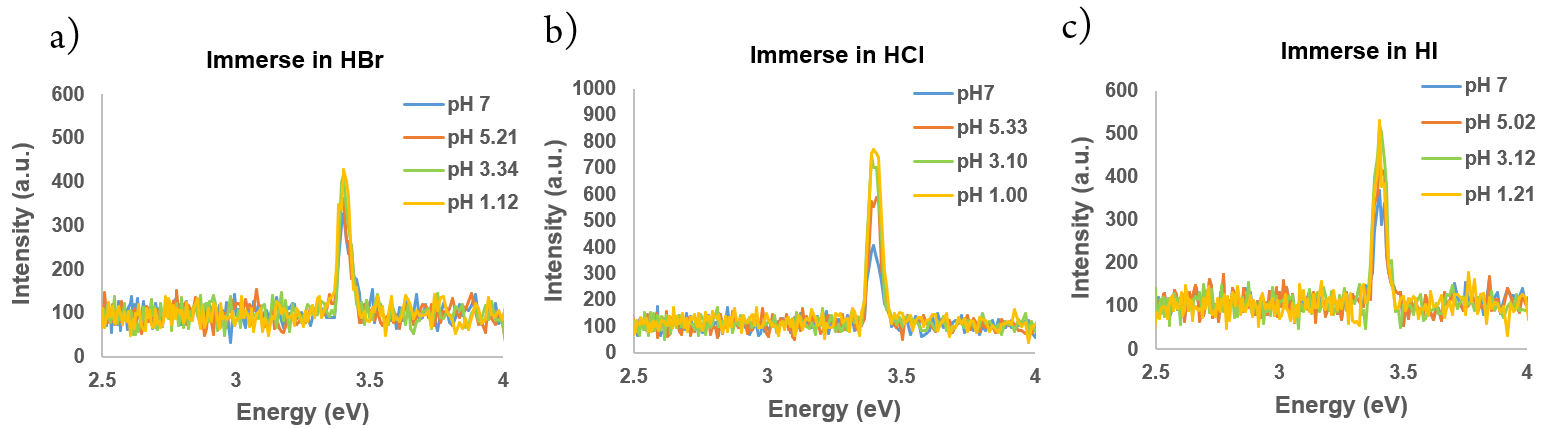


**Figure S7.** PL spectra when GaN nanowires are immersing in hydrohalic acids: (a) hydrobromic acid (HBr), (b) hydrochloric acid (HCl) and (c) hydriodic acid (HI).


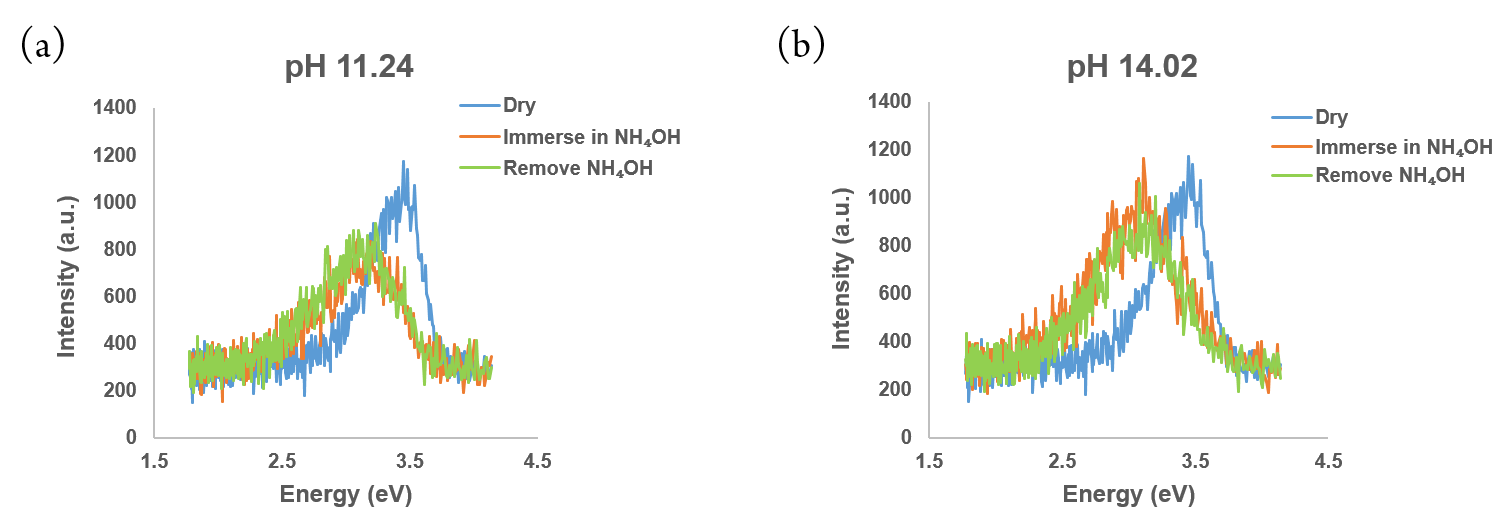


**Figure S8.** The PL spectra of GaN nanowires in NH₄OH at (a) pH=11.24, and (b) pH=14.02.
